# Use of sentinel lymph node biopsy in elderly patients with breast cancer – 10-year experience from a Swiss university hospital

**DOI:** 10.1186/s12957-023-03062-1

**Published:** 2023-06-08

**Authors:** Martin Heidinger, Nadia Maggi, Gilles Dutilh, Madleina Mueller, Ruth S. Eller, Julie M. Loesch, Fabienne D. Schwab, Christian Kurzeder, Walter P. Weber

**Affiliations:** 1 Breast Center, University Hospital Basel, Basel, Switzerland; 2 University of Basel, Basel, Switzerland; 3 Universitätsspital Basel, Spitalstrasse 21, 4031 Basel, Switzerland; 4 Department of Clinical Research, University Hospital Basel, Basel, Switzerland

**Keywords:** Breast cancer, Breast cancer surgery, Axillary surgery, Axillary staging, Sentinel lymph node biopsy, Elderly

## Abstract

**Background:**

The *Choosing Wisely* initiative recommended the omission of routine sentinel lymph node biopsy (SLNB) in patients ≥ 70 years of age, with clinically node-negative, early stage, hormone receptor (HR) positive and human epidermal growth factor receptor 2 (Her2) negative breast cancer in August 2016. Here, we assess the adherence to this recommendation in a Swiss university hospital.

**Methods:**

We conducted a retrospective single center cohort study from a prospectively maintained database. Patients ≥ 18 years of age with node-negative breast cancer were treated between 05/2011 and 03/2022. The primary outcome was the percentage of patients in the *Choosing Wisely* target group who underwent SLNB before and after the initiative went live. Statistical significance was tested using chi-squared test for categorical and Wilcoxon rank-sum tests for continuous variables.

**Results:**

In total, 586 patients met the inclusion criteria with a median follow-up of 2.7 years. Of these, 163 were ≥ 70 years of age and 79 were eligible for treatment according to the *Choosing Wisely* recommendations. There was a trend toward a higher rate of SLNB (92.7% vs. 75.0%, *p* = 0.07) after the *Choosing Wisely* recommendations were published. In patients ≥ 70 years with invasive disease, fewer received adjuvant radiotherapy after omission of SLNB (6.2% vs. 64.0%, *p* < 0.001), without differences concerning adjuvant systemic therapy. Both short-term and long-term complication rates after SLNB were low, without differences between elderly patients and those < 70 years.

**Conclusions:**

*Choosing Wisely* recommendations did not result in a decreased use of SLNB in the elderly at a Swiss university hospital.

**Supplementary Information:**

The online version contains supplementary material available at 10.1186/s12957-023-03062-1.

## Background

Sentinel lymph node biopsy (SLNB) currently represents the standard surgical staging procedure for the axilla in patients with clinically node-negative invasive breast cancer undergoing upfront surgery. It is a result of continuous efforts to de-escalate axillary surgery since the nineties [1–5], conferring to relevantly reduced morbidity when compared to axillary lymph node dissection (ALND) [1, 6, 7]; overall morbidity after SLNB is approximately 3–5% compared to 25% after ALND [1, 6–8]. In patients ≥ 70 years with stage I, hormone-receptor (HR) positive and human epidermal growth factor receptor 2 (Her2) negative breast cancer (BC), the routine role of SLNB was questioned, for example, by the CALGB 9343 trial, which showed no disadvantage in oncologic outcomes when surgical staging of the axilla was omitted [9]. SLNB is unlikely to inform systemic therapy choices in this patient population. It has therefore been deemed as low value [10]. *Choosing Wisely* recommendations of the Society of Surgical Oncology (SSO) have included the omission of routine usage of SLNB in these patients as of August 2016 [11]. However, rates of SLNB procedures in these patients remain high at 87–88% [12]. Arguments against the omission of SLNB in this subgroup comprise (i) uncertainty regarding the accuracy of age as procedure-specific cut-off value instead of patients‘ physiologic age (i.e., frailty status), (ii) categorization of SLNB as „low-risk “ procedure, (iii) promotion of patient peace of mind, (iv) staging information, and (v) absence of high-level evidence [13, 14]. In the present study, we investigate the routine use of SLNB with focus on the impact of the *Choosing Wisely* recommendation on clinical practice at a Swiss university breast center.

## Methods

We conducted a retrospective analysis of a prospectively maintained database. Patients with breast cancer of American Joint Committee on Cancer (AJCC) stage 0-IV underwent either breast conserving surgery (BCS) or mastectomy at the breast center of the University Hospital Basel from May 2011 to March 2022. Inclusion criteria included age ≥ 18 years, clinically negative nodal status and at least three months of follow-up. Clinically node-negative status was defined as unremarkable clinical examination including negative axillary palpation and ultrasound. In case of suspicious clinical findings, negative core-needle biopsy was mandatory. We included patients with DCIS because the use of the sentinel lymph node (SLN) procedure is indicated according to local standards in case of mastectomy, palpable or imaging-detected mass, diameter equal to or above 4 cm and potential histopathologic micro-invasion in the biopsy specimen. Surgical characteristics described the management of the axilla, which consisted of SLNB, tailored axillary surgery (TAS), ALND, or no axillary surgery. If neoadjuvant systemic therapy (NST) was administered and patients did undergo axillary surgery, the latter was performed after completion of NST. Baseline characteristics, treatment, and outcome variables were registered with the online good clinical practice conform clinical data management system secuTrial®, which is maintained by the Clinical Trial Unit Basel. Ethical approval was obtained from the local ethics committee (Ethikkommission Nordwest- und Zentralschweiz EKNZ, Approval number 2016–01525). Written informed consent was obtained from all participants.

### Outcomes

The outcome of interest was the percentage of patients who underwent SLNB by age (as surrogate for *Choosing Wisely* protocol eligibility) and time of treatment (before and after publication of *Choosing Wisely* recommendations). Secondary outcomes included influence of SLNB on adjuvant treatment decisions, as well as short-term (≤ 30 days) and long-term (> 30 days) complications after SLNB. Registered complications included delayed wound healing (defined as wound healing > 21 days), unplanned procedures as needle-aspiration or surgical revision (any surgery due to relevant hematoma, infection, abscess-formation, delayed wound-healing, fistulation), infection (necessitating either antibiotic treatment or surgical revision), clinically relevant seroma needing intervention (e.g., needle aspiration), axillary web syndrome, lymphedema, acute and chronic pain, and impairment of shoulder mobility. Complications were registered for the axilla and the breast.

### Sentinel lymph node biopsy

SLNB included all nodes that were either blue (blue dye) or hot (technetium Tc 99 m) or a combination thereof. Tc99m was applied into the dermis above the tumor the day before surgery, while blue dye was applied behind the nipple at the beginning of surgery, followed by massage for 5 min. Use of dual tracer was recommended during the earlier years of the study. Tc99m became the single tracer toward the end of the study without routine use of lymphoscintigraphy [15], and supported by blue dye injection selectively in case of weak signaling. The SLN procedure was performed either before, or after removal of the breast tumor. Whenever possible, the same incision was used both for SLNB and tumor removal. If this was not deemed appropriate at surgeons' discretion, a separate incision in the armpit of approximately 3–4 cm in the mid-axillary line, inferior to the lower axillary hairline-border was performed. Drainage systems were routinely placed on the chest wall after mastectomy, however an axillary drainage was not routinely placed. Skin closure was performed through intracutaneous resorbable suture.

### Statistical analysis

All analyses were accomplished on all available data from patients recorded in the secuTrial® database meeting the inclusion criteria. The primary research question (whether or not the frequency of SLNB treatment in the target group differed by age and time of treatment to reflect the impact of publication of the *Choosing Wisely* guidelines), was answered by performing a confirmatory Chi-squared test on the proportion of SLNB treatments before and after the recommendation’s publication, where the null-hypothesis constituted no change in this proportion. An alpha level of 5% was allowed. For a number of secondary outcomes, exploratory p-values are reported resulting from chi-squared tests for categorical and Wilcoxon rank-sum tests for continuous variables.

Descriptive statistics are reported for a range of patient and tumor characteristics, summarizing categorical variables as counts and percentages, and continuous variables by medians and quartile ranges. Statistical analysis was conducted using R (version 4.2.2) [16].

## Results

We included 586 patients with clinically negative nodal status over the study period. Median follow-up was 2.7 years. Median age was 61 years (interquartile range (IQR) 51–71), with 98.0% being Caucasian. Most tumors were clinically stage T1 (53.4%), invasive ductal carcinomas (IDC) (63.7%), HR positive and Her2 negative (82.8%), without lymphovascular invasion (L0) (82.3%), and grade 2 (49.3%; Table 1).

**Table 1:** Patient and tumor characteristics

	**Total** **(*n* = 586)**	** < 70 years** **(*n* = 419)**	** ≥ 70 years** **(*n* = 163)**	***p*-value**	% missing data
**Age (median [IQR])**	61.0 [51.0,71.0]	55.0 [48.0, 62.0]	76.0 [73.0, 80.5]	< 0.001	0.7%
**Ethnicity**				0.296	6.5%
Caucasian	537 (98.0%)	384 (97.5%)	149 (99.3%)		
Other	11 (2.0%)	10 (2.5%)	1 (0.7%)		
**Clinical tumor stage**				0.015	0.0%
DCIS	90 (15.4%)	76 (18.1%)	14 (8.6%)		
T1	307 (53.4%)	215 (51.3%)	90 (55.2%)		
T2	177 (30.2%)	121 (28.9%)	54 (33.1%)		
T3	8 (1.4%)	6 (1.4%)	2 (1.2%)		
T4	4 (0.7%)	1 (0.2%)	3 (1.8%)		
**Histological type**				0.019	1.2%
DCIS	98 (16.9%)	80 (19.3%)	18 (11.2%)		
Invasive ductal	369 (63.7%)	265 (63.9%)	100 (62.5%)		
Invasive lobular	55 (9.5%)	33 (8.0%)	22 (13.8%)		
Other	57 (9.8%)	37 (8.9%)	20 (12.5%)		
**Receptor status**				0.163	3.6%
HR + , Her2 -	486 (82.8%)	329 (80.8%)	136 (88.3%)		
HR + , Her2 +	42 (7.4%)	35 (8.6%)	6 (3.9%)		
HR -, Her2 +	16 (2.8%)	12 (2.9%)	4 (2.6%)		
Triple negative	39 (6.9%)	31 (7.6%)	8 (5.3%)		
**Tumor grade**				0.005	6.8%
Grade 1	142 (26.0%)	109 (27.5%)	32 (21.9%)		
Grade 2	269 (49.3%)	178 (44.9%)	88 (60.3%)		
Grade 3	135 (24.7%)	109 (27.5%)	26 (17.8%)		
**Lymphovascular invasion**				0.013	10.4%
L0	432 (82.3%)	321 (85.1%)	111 (75.5%)		
L1	93 (17.7%)	56 (14.9%)	36 (24.5%)		
**Type of axillary surgery**				0.307	4.6%
Sentinel lymph node biopsy	442 (79.1%)	317 (79.6%)	122 (77.7%)		
No axillary surgery	71 (12.7%)	47 (11.8%)	23 (14.6%)		
Tailored axillary surgery^a^	17 (3.0%)	15 (3.8%)	2 (1.3%)		
Axillary lymph node dissection	29 (5.2%)	19 (4.8%)	10 (6.4%)		
**Type of breast surgery**					4.9%
Breast conserving surgery	386 (69.3%)	272 (68.7%)	111 (70.7%)	0.718	
Mastectomy	171 (30.7%)	124 (31.3%)	46 (29.3%)	0.732	
**Pathological nodal status**				0.578	9.6%
pNx	45 (8.5%)	28 (7.5%)	16 (10.3%)		
pN0	396 (74.7%)	281 (75.7%)	112 (72.3%)		
pN1	77 (14.5%)	54 (14.6%)	23 (14.8%)		
pN2	6 (1.1%)	5 (1.3%)	1 (0.6%)		
pN3	6 (1.1%)	3 (0.8%)	3 (1.9%)		

Percentages add up to 100% in each age group. Note that the overall totals may differ from the sum of n for both age groups, due to missing age information for some patients. For categorical variables, p-values are based on χ2 tests, where the null-hypothesis states that the distribution across the levels of the relevant factor is equal across age groups. For continuous variables, p-values are based on Wilcoxon rank-sum test, where the null hypothesis states that the distribution of that variable is equal across age groups

*IQR* interquartile range, *DCIS* ductal carcinoma in situ, *HR* hormone receptor, *Her2* human epidermal growth factor receptor 2

^a^Tailored axillary surgery consists of the removal of sentinel lymph nodes, palpably suspicious findings, as well as the optional selective removal of lymph nodes localized under image-guidance [17]

SLNB was performed in 442 patients (79.1%). The median number of retrieved nodes using SLNB was 2 (IQR 1–3), with 12.2% (54/442) having at least one positive SLN on final histopathological examination. Of those patients with SLN metastases, 11 (20.4%) went on to receive completion axillary lymph node dissection (cALND) in a second surgery.

The cohort of patients ≥ 70 years comprised of 163 individuals, with a median age of 76 years (IQR 73.0–80.5). Most had T1 (55.2%), IDC (62.5%), HR positive and Her2 negative (88.3%), grade 2 (60.3%) tumors. The majority received SLNB (77.7%), whereas a median of 2 lymph nodes were retrieved (IQR 1–3) during SLNB, with 13.1% (16/122) showing at least one involved SLN. Of those patients with SLN metastases, five (31.3%) went on to receive cALND.

Neoadjuvant and adjuvant treatment differed by age (Table 2). Chemotherapy and adjuvant radiotherapy were applied more frequently in younger patients. In the subgroup of patients with HR positive and Her2 negative BC, adjuvant radiotherapy was more frequently used in patients < 70 years of age. Antihormonal therapy was administered in 82.0% of patients with HR positive and Her2 negative BC, without apparent differences between subgroups.

**Table 2:** Rates of neoadjuvant and adjuvant therapies in the total cohort of patients with clinically node-negative breast cancer and those with hormone receptor positive, human epidermal growth factor receptor 2 negative breast cancer

**Total cohort**	**< 70 years** **(*n*** **= 419)**	**≥ 70 years** **(*n*** **= 163)**	***p*-value**	**% missing data**
Neoadjuvant chemotherapy	34 (8.2%)	2 (1.2%)	0.003	1.0
Neoadjuvant anti-hormonal therapy	15 (3.6%)	9 (5.5%)	0.429	1.0
Adjuvant chemotherapy	74 (17.9%)	17 (10.8%)	0.051	1.7
Adjuvant anti-hormonal therapy	307 (74.5%)	120 (75.0%)	0.99	1.7
Adjuvant radiotherapy	280 (67.6%)	88 (55.0%)	0.006	1.4
**HR + , Her2-**	**< 70 years** **(*n*** **= 329)**	**≥ 70 years** **(*n*** **= 136)**	***p*-value**	**% missing data**
Neoadjuvant chemotherapy	7 (2.2%)	0 (0.0%)	0.191	5.1
Neoadjuvant anti-hormonal therapy	14 (4.3%)	8 (5.9%)	0.629	5.1
Adjuvant chemotherapy	44 (13.5%)	10 (7.6%)	0.111	5.9
Adjuvant anti-hormonal therapy	262 (80.9%)	113 (84.3%)	0.458	5.7
Adjuvant radiotherapy	222 (68.1%)	73 (54.5%)	0.008	5.3

Percentages per category do not take missing values into account

*HR* hormone receptor, *Her2* human epidermal growth factor receptor 2

To answer our primary research question, we compared the rate of SLNB before and after publication of the *Choosing Wisely* recommendations (August 2016) for the target group of patients, who are ≥ 70 years, with cT1, cN0, HR positive and Her2 negative breast cancer. There was an increase of SLNBs from 75.0% to 92.7% (*p* = 0.07) after the publication of *Choosing Wisely* recommendations (Table 3). Metastasis in the SLN were found in 13.0% (9/69) of patients in the target group, with 44.4% (4/9) receiving cALND. We studied the possibility that SLNB procedures were confounded by across-time differences in tumor characteristics and treatment decisions. Neither tumor grade, neoadjuvant treatment, nor type of breast surgery could be identified as such confounders (Supplementary Table 1).

**Table 3:** Rates of axillary surgery in patients with cT1, cN0, hormone receptor positive, human epidermal growth factor receptor 2 negative breast cancer until and after August 2016

	** < 70 years**		** ≥ 70 years**	
	**Until August 2016** **(*n* = 64)**	**As of August 2016** **(*n* = 97)**	**Until August 2016** **(*n* = 24)**	**As of August 2016** **(*n* = 55)**
**Sentinel lymph node biopsy** **(n total = 213)**	89.1%	89.7%	75.0%	92.7%
**No axillary surgery** **(n total = 12)**	4.7%	4.1%	8.3%	5.5%
**Tailored axillary surgery**^a^ **(n total = 5)**	3.1%	3.1%	0.0%	0.0%
**Axillary lymph node dissection (n total = 10)**	3.1%	3.1%	16.7%	1.8%

^a^Tailored axillary surgery consists of the removal of sentinel lymph nodes, palpably suspicious findings, as well as the optional selective removal of lymph nodes localized under image-guidance [17]

Furthermore, patients in the target group exhibited a decrease in omission of axillary surgery from 8.3% to 5.5%. The rate of ALND in the target group appeared to decrease from 16.7% before 08/2016, to 1.8% thereafter. In patients < 70 years the rates of axillary surgery appeared to remain stable between the two periods as shown in Table 3.

Next, we compared treatment approaches in patients ≥ 70 years with invasive disease (*n* = 136). Breast conserving surgery was performed in 50% of patients in whom SLNB was omitted (*n* = 16) with the remaining 50% undergoing mastectomy. Patients with SLNB (*n* = 116) more frequently underwent BCS (76.7%; *p* = 0.049). There was no difference regarding adjuvant chemotherapy (6.7% vs. 9.7%, *p* = 1.0) and anti-hormonal therapy (75.0% vs. 78.9%, *p* = 0.972). However, adjuvant radiotherapy was conducted less frequently in patients in whom SLNB was omitted (6.2% vs. 64.0%, p < 0.001).

The rate of SLNB in patients with DCIS decreased numerically in the total cohort by time of treatment (67.7% in early vs. 49.1% in late period, *p* = 0.15). Whilst a numerical reduction of SLNB in patients with BCS was observed (40.0% vs. 27.0%, *p* = 0.68), patients undergoing mastectomy had continuously high rates of SLNB (85.0% vs. 94.4%, *p* = 0.68).

Both short-term and long-term axillary complication rates of patients receiving SLNB were similar for patients < 70 years and the elderly as depicted in Table 4.

**Table 4:** Short-term (< 30 days) and long-term (≥ 30 days) complication rates in patients receiving sentinel lymph node biopsy

	** < 70 years** **(*n* = 311**^**b**^**)**	** ≥ 70 years** **(*n* = 117**^**b**^**)**
**Short-term complications**		
Any complication	46 (14.8%)	18 (15.4%)
Surgical revision ^a^	8 (2.6%)	1 (0.9%)
Infection ^a^	10 (3.2%)	3 (2.6%)
Axillary infection	2 (0.6%)	0 (0.0%)
Clinically relevant seroma ^a^	14 (4.5%)	5 (4.3%)
Clinically relevant axillary seroma	4 (1.3%)	3 (2.6%)
**Long-term complications**		
Any complication	98 (31.5%)	30 (25.6%)
Clinically relevant seroma ^a^	11 (3.5%)	5 (4.3%)
Clinically relevant axillary seroma	4 (1.3%)	3 (2.6%)
Axillary web syndrome	6 (1.9%)	0 (0.0%)
Lymphedema	10 (3.2%)	3 (2.6%)
Chronic pain ^a^	69 (22.2%)	17 (14.5%)
Chronic axillary pain	26 (8.4%)	8 (6.8%)
Chronic arm pain	14 (4.5%)	1 (0.9%)
Impairment of shoulder mobility	20 (6.4%)	5 (4.3%)

^a^Including any site (breast, axilla); clinically relevant being defined as needing intervention

^b^Excluding patients who received completion axillary lymph node dissection in a second surgery

## Discussion

The *Choosing Wisely* campaign was developed to reduce low value surgical procedures with the aim of de-implementing potentially harmful and costly procedures that do not improve survival. In August 2016, the initiative recommended to omit routine use of SLNB in women ≥ 70 years with clinically node-negative early stage HR positive and Her2 negative BC [11], which is not the main cause of death in those patients [9]. Furthermore, the SLN procedure is questioned as it is neither causing a survival benefit, nor a clinically relevant benefit in locoregional control. Moreover, a clear indication for adjuvant endocrine therapy is given, irrespective of nodal status [9, 18].

The present retrospective cohort study aimed to investigate the impact of the *Choosing Wisely* recommendations on clinical practice at a Swiss university hospital. It showed that SLNB did not decrease in elderly patients with small clinically node-negative BC after the publication of these recommendations. This is in line with findings from a retrospective US multi-center study that showed institutional variation ranging from 25%-97%, with overall stable use of SLNB in *the Choosing Wisely* group before and after its publication (88% in 2013, and 87% in 2016), while the later period may have been too early to detect any impact of the recommendations on clinical practice [12]. Earlier real-world data clearly showed routine axillary staging by SLNB in the elderly [19].

The present study showed that the omission of SLNB in elderly BC patients was not associated with an increase in the use of adjuvant radiotherapy of the breast. In fact, the fraction of patients undergoing radiotherapy was significantly smaller when SLNB was omitted. Whilst this is in line with the study protocol of the CALGB 9343 trial, applicability of the other landmark trial protocol concerning the omission of radiotherapy in elderly BC patients—PRIME II—depended on surgically confirmed negative nodal status of the axilla [9, 18, 20, 21]. Surgical axillary staging was also mandatory in the majority of trials laying the foundation for a recent European consensus recommendation on hypofractionated radiotherapy and partial breast irradiation [22]. The Florence trial being the only exception, including however only 23 patients without axillary surgery, currently preventing any evidence-based conclusions [23]. Therefore, interdisciplinary consensus should be sought in the tumor boards that de-escalation of surgical staging of the axilla does not result in overuse of adjuvant radiotherapy of the breast and axilla. A recent Canadian population-based study has shown lower rates of adjuvant radiotherapy to the breast, however higher rates of axillary radiotherapy in patients ≥ 70 years with stage I or II BC after omission of surgical axillary staging [24]. In earlier studies, axillary surgery was found to be associated with adjuvant therapy in the elderly [19, 25, 26]. Nevertheless, whilst the rate of neoadjuvant and adjuvant chemotherapy, as well as adjuvant radiotherapy in our general population showed age-dependent differences, omission of axillary surgery did not differ by age. Interestingly, ALND showed a numerical decrease in the elderly after August 2016, potentially representing the influence of the Z0011 trial on clinical practice [4].

What is the potential benefit of SLNB omission? SLNB-associated morbidity is still considered relevant especially in the elderly with significant co-morbidities. Subjective arm and shoulder morbidity is reported to be present in one-quarter of patients one week after undergoing SLNB, according to results from the SOUND trial [27]. Similar findings were recently shown in the quality of life report of the INSEMA study [28]. Compound arm morbidity one year after SLNB, including arm swelling, lymphedema, pain, paresthesia, and decreased shoulder mobility, was reported in the OTOASOR trial as being 4.7% [6]. SLNB has been associated with lymphedema in 1–15% in cohorts receiving axillary radiotherapy (2.6% in our elderly-cohort) [1, 5–7]. Furthermore, short-term shoulder mobility impairment is reported (4.3% in our elderly-cohort), and chronic pain in 1–7% (14.5% in our elderly-cohort, with 6.8% reporting chronic axillary pain, and 0.9% chronic arm pain) [1, 7]. These results also hold true for women with DCIS undergoing SLNB [29]. Despite guidelines discouraging the use of SLNB in DCIS patients undergoing BCS, result from the US have shown an increase of SLNB in these patients between 2005–2017 to 20.9%-22.8% corresponding to the reported rates in our study of 27–40% [30]. In summary, arm morbidity may cause functional impairment with reduced autonomy in activities of daily living, particularly in the earliest postoperative period, potentially also aggravating underlying conditions.

However, nodal status is still the most important prognostic factor and needed for local as well as adjuvant treatment decisions. De-escalating and tailoring axillary surgery has seen major developments from the Halstedian radical mastectomy, to standard ALND [31] and SLNB [1–3], as well as ALND omission in clinically node-negative, SLN positive BC patients [4, 5]. ALND is an accurate staging procedure, but causes much morbidity [1, 6–8, 32, 33]. SLNB is standard of care in axillary staging and remains primarily a diagnostic procedure. A relevant therapeutic potential of SLNB has been suggested when omission of ALND in clinically node-negative BC patients with positive SLNs was shown to be safe. Importantly, residual nodal disease after SLNB was found in 27%, 33%, and 44% in the Z0011, AMAROS, and SINODAR trials respectively, which did not translate into worse oncologic outcomes [4, 5, 34].

Finally, the omission of any axillary surgery is not a new paradigm. Several trials randomized patients with clinically node-negative BC to omission of ALND without showing worse oncologic outcomes [31, 35–38]. The *Choosing Wisely* recommendations to omit SLNB in elderly patients with luminal BC was primarily inspired by the CALGB 9343 trial, which randomized elderly patients to receive tamoxifen with or without radiotherapy, with 62% of patients forgoing any axillary surgery [9, 21]. Of those, none experienced an axillary recurrence in the group with radiotherapy, compared to six patients without radiotherapy after a median follow-up of 12.6 years. In the general study population, no significant differences in overall survival, breast-cancer specific survival, time to distant metastases, and time to mastectomy were noted. Of the recorded deaths in the study population, only 6.7% were breast cancer related [9]. Recently, a nomogram was developed for selective omission of SLNB. It is based on age, cN0, histologic subtype, tumor grade, multifocality, and tumor size. The calculated false-negative rate of 5% for macrometastatic disease would allow one-third of patients to safely forego SLNB [39].

Axillary imaging has traditionally been considered not accurate enough to stage the axilla by itself and consists primarily of ultrasound, which showed a positive-predictive value of 58–81% and a negative predictive value of 71–79% [40–42]. Even though imperfect when used alone, axillary ultrasound helps refine SLN positivity prediction when incorporated into a nomogram [43]. A negative ultrasound was also the main eligibility criterion for patients to enter randomized trials that investigated the use of SLNB in contemporary patients with low risk early breast cancer [44–48]. Pending results have the potential to change practice toward complete de-escalation of axillary surgery in many patients with negative ultrasound. Even though some of these trials are restricted to candidates for BCS, most of them include cancers up to 5 cm, all age groups, and all intrinsic subtypes, and the benefit of SLNB in patients undergoing mastectomy is increasingly questioned [49]. As results of these potentially practice changing trials are eagerly expected, it will be important to assess their impact on surgical clinical practice, adjuvant treatments and oncologic outcomes also by intrinsic subtypes in subsequent implementation studies. Finally, the question pertains whether a more sophisticated examination including comorbidities and life-expectancy as well as functional capacities within e.g., a comprehensive geriatric assessment should play a stronger role in selection of patients for omission of SLNB [50, 51]. As long as SLNB remains standard care in most women with clinically node-negative invasive breast cancer, morbidity should be minimized by adequate training of the next generation of breast surgeons. Quality indicators could be based on minimum caseload and reflected by quality assurance and certification programs.

### Limitations

The main limitation of our study is the retrospective, single center, observational design, which carries an inherent potential for selection bias. Our analysis does not account for co-morbidities as potential confounding factors. In addition, the sample size was limited, making larger prospective datasets necessary to validate our findings.

## Conclusions

*Choosing Wisely* recommendations to omit SLNB in patients ≥ 70 years with small, clinically node-negative, luminal breast cancer were not followed at a Swiss university hospital. Underlying factors need to be identified to prepare for incorporation of pending results from SLNB omission trials into clinical practice.

## Supplementary Information

**Additional file 1: Supplement Table 1.** Comparison of potential influence factors on axillary surgery in patients ≥70 years, with cT1, cN0, hormone receptor positive, human epidermal growth factor receptor 2 negative breast cancer before and after publication of Choosing Wisely recommendations.

## Data Availability

All data generated or analyzed during this study are included in this published article and its supplementary information files.

## References

[1] Veronesi U, Paganelli G, Viale G (2003). A randomized comparison of sentinel-node biopsy with routine axillary dissection in breast cancer. N Engl J Med.

[2] Krag DN, Anderson SJ, Julian TB (2007). Technical outcomes of sentinel-lymph-node resection and conventional axillary-lymph-node dissection in patients with clinically node-negative breast cancer: results from the NSABP B-32 randomised phase III trial. Lancet Oncol.

[3] Krag DN, Anderson SJ, Julian TB (2010). Sentinel-lymph-node resection compared with conventional axillary-lymph-node dissection in clinically node-negative patients with breast cancer: Overall survival findings from the NSABP B-32 randomised phase 3 trial. Lancet Oncol.

[4] Giuliano AE, Ballman KV, McCall L (2017). Effect of axillary dissection vs no axillary dissection on 10-year overall survival among women with invasive breast cancer and sentinel node metastasis: The ACOSOG Z0011 (Alliance) randomized clinical trial. JAMA.

[5] Bartels SAL, Donker M, Poncet C (2022). Radiotherapy or Surgery of the Axilla After a Positive Sentinel Node in Breast Cancer: 10-Year Results of the Randomized Controlled EORTC 10981–22023 AMAROS Trial. J Clin Oncol.

[6] Sávolt PG, Polgár C (2017). Eight-year follow up result of the OTOASOR trial: The Optimal Treatment Of the Axilla – Surgery Or Radiotherapy after positive sentinel lymph node biopsy in early-stage breast cancer: A randomized, single centre, phase III, non-inferiority trial. Eur J Surg Oncol.

[7] Mansel RE, Fallowfield L, Kissin M (2006). Randomized multicenter trial of sentinel node biopsy versus standard axillary treatment in operable breast cancer: the ALMANAC Trial. J Natl Cancer Inst.

[8] Armer JM, Ballman KV, McCall L (2019). Factors Associated with Lymphedema in Women with Node-Positive Breast Cancer Treated with Neoadjuvant Chemotherapy and Axillary Dissection. JAMA Surg.

[9] Hughes KS, Schnaper LA, Bellon JR (2013). Lumpectomy plus tamoxifen with or without irradiation in women age 70 years or older with early breast cancer: Long-term follow-up of CALGB 9343. J Clin Oncol.

[10] Shubeck SP, Morrow M, Dossett LA. De-escalation in breast cancer surgery. NPJ Breast Cancer. 2022; 8. 10.1038/s41523-022-00383-4. 10.1038/s41523-022-00383-4 PMC8866473 35197478

[11] Sentinel node biopsy | Choosing Wisely. https://www.choosingwisely.org/clinician-lists/sso-sentinel-node-biopsy-in-node-negative-women-70-and-over/. Accessed 26 Feb 2023.

[12] Wang T, Bredbeck BC, Sinco B (2021). Variations in Persistent Use of Low-Value Breast Cancer Surgery. JAMA Surg.

[13] Smith ME, Vitous CA, Hughes TM (2020). Barriers and Facilitators to De-Implementation of the Choosing Wisely® Guidelines for Low-Value Breast Cancer Surgery. Ann Surg Oncol.

[14] Wang T, Mott N, Miller J (2020). Patient Perspectives on Treatment Options for Older Women with Hormone Receptor-Positive Breast Cancer: A Qualitative Study. JAMA Netw Open.

[15] Kuemmel S, Holtschmidt J, Gerber B (2019). Prospective, multicenter, randomized phase III trial evaluating the impact of lymphoscintigraphy as part of sentinel node biopsy in early breast cancer: Senszi (GBG80) trial. J Clin Oncol.

[16] R Core Team (2022). R: A language and environment for statistical computing.

[17] Weber WP, Matrai Z, Hayoz S (2021). Tailored axillary surgery in patients with clinically node-positive breast cancer: Pre-planned feasibility substudy of TAXIS (OPBC-03, SAKK 23/16, IBCSG 57–18, ABCSG-53, GBG 101). Breast.

[18] Kunkler IH, Williams LJ, Jack WJL (2015). Breast-conserving surgery with or without irradiation in women aged 65 years or older with early breast cancer (PRIME II): A randomised controlled trial. Lancet Oncol.

[19] Dominici LS, Sineshaw HM, Jemal A (2018). Patterns of axillary evaluation in older patients with breast cancer and associations with adjuvant therapy receipt. Breast Cancer Res Treat.

[20] Kunkler IH, Williams LJ, Jack WJL (2023). Breast-Conserving Surgery with or without Irradiation in Early Breast Cancer. N Engl J Med.

[21] Hughes KS, Schnaper LA, Berry D (2004). Lumpectomy plus Tamoxifen with or without Irradiation in Women 70 Years of Age or Older with Early Breast Cancer. N Engl J Med.

[22] Meattini I, Becherini C, Boersma L (2022). European Society for Radiotherapy and Oncology Advisory Committee in Radiation Oncology Practice consensus recommendations on patient selection and dose and fractionation for external beam radiotherapy in early breast cancer. Lancet Oncol.

[23] Livi L, Meattini I, Marrazzo L (2015). Accelerated partial breast irradiation using intensity-modulated radiotherapy versus whole breast irradiation: 5-year survival analysis of a phase 3 randomised controlled trial. Eur J Cancer.

[24] Castelo M, Sutradhar R, Faught N, et al. The Association Between Surgical Axillary Staging, Adjuvant Treatment Use and Survival in Older Women with Early Stage Breast Cancer: A Population-Based Study. Ann Surg Oncol. 2023; 10.1245/s10434-023-13274-0. 10.1245/s10434-023-13274-0 36917335

[25] Chagpar AB, Horowitz N, Sanft T (2017). Does lymph node status influence adjuvant therapy decision-making in women 70 years of age or older with clinically node negative hormone receptor positive breast cancer?. Am J Surg.

[26] Tamirisa N, Thomas SM, Fayanju OM (2018). Axillary Nodal Evaluation in Elderly Breast Cancer Patients: Potential Effects on Treatment Decisions and Survival. Ann Surg Oncol.

[27] Gentilini O, Botteri E, Dadda P (2016). Physical function of the upper limb after breast cancer surgery. Results from the SOUND (Sentinel node vs. Observation after axillary Ultra-souND) trial. Eur J Surg Oncol.

[28] Reimer T, Stachs A, Veselinovic K, et al. Patient-reported outcomes for the Intergroup Sentinel Mamma study (INSEMA): A randomised trial with persistent impact of axillary surgery on arm and breast symptoms in patients with early breast cancer. eClinicalMedicine. 2023; 55:101756. 10.1016/j.eclinm.2022.101756. 10.1016/j.eclinm.2022.101756 PMC9706517 36457648

[29] Killelea BK, Long JB, Dang W (2018). Associations Between Sentinel Lymph Node Biopsy and Complications for Patients with Ductal Carcinoma In Situ. Ann Surg Oncol.

[30] Pyfer BJ, Jonczyk M, Jean J (2020). Analysis of Surgical Trends for Axillary Lymph Node Management in Patients with Ductal Carcinoma In Situ Using the NSQIP Database: Are We Following National Guidelines?. Ann Surg Oncol.

[31] Fisher B, Redmond C, Fisher ER (1985). Ten-Year Results of a Randomized Clinical Trial Comparing Radical Mastectomy and Total Mastectomy with or without Radiation. N Engl J Med.

[32] Ashikaga T, Krag DN, Land SR (2010). Morbidity results from the NSABP B-32 trial comparing sentinel lymph node dissection versus axillary dissection. J Surg Oncol.

[33] Land SR, Kopec JA, Julian TB (2010). Patient-reported outcomes in sentinel node-negative adjuvant breast cancer patients receiving sentinel-node biopsy or axillary dissection: National Surgical Adjuvant Breast and Bowel Project phase III protocol B-32. J Clin Oncol.

[34] Tinterri C, Gentile D, Gatzemeier W (2022). Preservation of Axillary Lymph Nodes Compared with Complete Dissection in T1–2 Breast Cancer Patients Presenting One or Two Metastatic Sentinel Lymph Nodes: The SINODAR-ONE Multicenter Randomized Clinical Trial. Ann Surg Oncol.

[35] Louis-Sylvestre C, Clough K, Asselain B (2004). Axillary treatment in conservative management of operable breast cancer: Dissection or radiotherapy? Results of a randomized study with 15 years of follow-up. J Clin Oncol.

[36] Agresti R, Martelli G, Sandri M (2014). Axillary lymph node dissection versus no dissection in patients with T1N0 breast cancer: A randomized clinical trial (INT09/98). Cancer.

[37] Martelli G, Boracchi P, Ardoino I (2012). Axillary Dissection Versus No Axillary Dissection in Older Patients With T1N0 Breast Cancer 15-Year Results of a Randomized Controlled Trial. Ann Surg.

[38] Rudenstam C-M, Zahrieh D, Forbes J (2006). Randomized Trial Comparing Axillary Clearance Versus No Axillary Clearance in Older Patients With Breast Cancer: First Results of International Breast Cancer Study Group Trial 10–93. J Clin Oncol.

[39] Moorman AM, Rutgers EJT, Kouwenhoven EA (2022). Omitting SLNB in Breast Cancer: Is a Nomogram the Answer?. Ann Surg Oncol.

[40] Riedel F, Schaefgen B, Sinn HP, et al. Diagnostic accuracy of axillary staging by ultrasound in early breast cancer patients. Eur J Radiol. 2021; 135. 10.1016/j.ejrad.2020.109468. 10.1016/j.ejrad.2020.109468 33338758

[41] Ertan K, Linsler C, di Liberto A (2013). Axillary ultrasound for breast cancer staging: An attempt to identify clinical/histopathological factors impacting diagnostic performance. Breast Cancer Basic Clin Res.

[42] Leenders MWH, Broeders M, Croese C (2012). Ultrasound and fine needle aspiration cytology of axillary lymph nodes in breast cancer. To do or not to do?. Breast.

[43] Tran HT, Pack D, Mylander C (2020). Ultrasound-Based Nomogram Identifies Breast Cancer Patients Unlikely to Harbor Axillary Metastasis: Towards Selective Omission of Sentinel Lymph Node Biopsy. Ann Surg Oncol.

[44] Sentinel Node Vs Observation After Axillary Ultra-souND (SOUND). https://www.clinicaltrials.gov/ct2/show/NCT02167490. Accessed 7 Jan 2023.

[45] Comparison of Axillary Sentinel Lymph Node Biopsy Versus no Axillary Surgery (INSEMA). https://clinicaltrials.gov/ct2/show/NCT02466737. Accessed 7 Jan 2023.

[46] Jung JG, Ahn SH, Lee S, et al. No axillary surgical treatment for lymph node-negative patients after ultra-sonography [NAUTILUS]: protocol of a prospective randomized clinical trial. BMC Cancer. 2022; 22. 10.1186/s12885-022-09273-1. 10.1186/s12885-022-09273-1 PMC8859876 35184724

[47] van Roozendaal LM, Vane MLG, van Dalen T, et al. Clinically node negative breast cancer patients undergoing breast conserving therapy, sentinel lymph node procedure versus follow-up: A Dutch randomized controlled multicentre trial (BOOG 2013–08). BMC Cancer. 2017; 17. 10.1186/s12885-017-3443-x. 10.1186/s12885-017-3443-x PMC5494134 28668073

[48] Sentinel Node Biopsy Vs Observation After Axillary PET - Full Text View - ClinicalTrials.gov. https://clinicaltrials.gov/ct2/show/NCT04072653. Accessed 20 Feb 2023.

[49] Matar R, Barrio AV, Sevilimedu V (2022). Can We Successfully De-Escalate Axillary Surgery in Women Aged ≥ 70 Years with Ductal Carcinoma in Situ or Early-Stage Breast Cancer Undergoing Mastectomy?. Ann Surg Oncol.

[50] Williams GR, Jones E, Muss HB (2013). Challenges in the treatment of older breast cancer patients. Hematol Oncol Clin North Am.

[51] Kim Dittus MD, PhD;Hyman B. Muss M,  (2007). Management of the Frail Elderly With Breast Cancer. Oncology.

