# Supplementary material for: Use of sentinel lymph node biopsy in elderly patients with breast cancer – 10-year experience from a Swiss university hospital
Source: World J Surg Oncol. 2023 Jun 8;21:176. doi: 10.1186/s12957-023-03062-1 (PMC10249276; doi:10.1186/s12957-023-03062-1)
Supplement: Supplementary file 1 — Additional file 1: Supplement Table 1. Comparison of potential influence factors on axillary surgery in patients ≥70 years, with cT1, cN0, hormone receptor positive, human epidermal growth factor receptor 2 negative breast cancer before and after publication of Choosing Wisely recommendations. [file 12957_2023_3062_MOESM1_ESM.docx]

# Supplementary Information

# Use of sentinel lymph node biopsy in elderly patients with breast cancer – 10-year experience from a Swiss university hospital

Martin Heidinger†* ^1,2^, Nadia Maggi* ^1,2^, Gilles Dutilh ^3^, Madleina Mueller ^1^, Ruth S. Eller ^1^, Julie M. Loesch ^1^, Fabienne D. Schwab ^1,2^, Christian Kurzeder ^1^, Walter P. Weber ^1,2^

^1^ Breast Center, University Hospital Basel, Basel, Switzerland

^2^ University of Basel, Basel, Switzerland

^3^ Department of Clinical Research, University Hospital Basel, Basel, Switzerland

* shared first co-authorship – contributed equally

† corresponding author

Dr. Martin Heidinger

[martin.heidinger@usb.ch](mailto:martin.heidinger@usb.ch)

Brustchirurgie, Universitätsspital Basel

Spitalstrasse 21, 4031 Basel

**Supplement Table 1**. Comparison of potential influence factors on axillary surgery in patients ≥70 years, with cT1, cN0, hormone receptor positive, human epidermal growth factor receptor 2 negative breast cancer before and after publication of *Choosing Wisely* recommendations

|  | Until August 2016  (n=24) | As of August 2016  (n=55) | p-value | %  missing data |
| --- | --- | --- | --- | --- |
| **Neoadjuvant therapy** | 0.0% | 1.8% | 1.0 | 4.8% |
| **Tumor grade** | | | | 9.6% |
| Grade 1 | 26.1% | 23.1% | 0.39 |  |
| Grade 2 | 56.5% | 69.2% |  |  |
| Grade 3 | 17.4% | 7.7% |  |  |
| **Type of breast surgery** | | | | 4.8% |
| Breast conserving surgery | 87.5% | 70.9% | 0.19 |  |
| Mastectomy | 12.5% | 29.1% |  |  |
